# Study on the expression of TOP2A in hepatocellular carcinoma and its relationship with patient prognosis

**DOI:** 10.1186/s12935-021-02439-0

**Published:** 2022-01-15

**Authors:** Jiali Meng, Yuanchao Wei, Qing Deng, Ling Li, Xiaolong Li

**Affiliations:** 1 Clinical Medicine, Guangxi Medical University, Nanning, Guangxi China; 2 Department of Cell Biology and Genetics, School of Pre-Clinical Medicine, Key Laboratory of Longevity and Agingrelated Diseases of Chinese Ministry of Education, Guangxi Medical University, Nanning, 530021 China; 3 Department of Pathology, The People’s Hospital of Guangxi Zhuang Autonomous Region, Nanning, China

**Keywords:** HCC, TOP2A, Bioinformatics, Immunohistochemical test

## Abstract

**Background:**

Hepatocellular carcinoma (HCC) is a primary liver cancer with a high mortality rate. However, the molecular mechanism of HCC formation remains to be explored and studied.

**Objective:**

To investigate the expression of TOP2A in hepatocellular carcinoma (HCC) and its prognosis.

**Methods:**

The data set of hepatocellular carcinoma was downloaded from GEO database for differential gene analysis, and hub gene was identified by Cytoscape. GEPIA was used to verify the expression of HUB gene and evaluate its prognostic value. Then TOP2A was selected as the research object of this paper by combining literature and clinical sample results. Firstly, TIMER database was used to study TOP2A, and the differential expression of TOP2A gene between normal tissues and cancer tissues was analyzed, as well as the correlation between TOP2A gene expression and immune infiltration of HCC cells. Then, the expression of top2a-related antibodies was analyzed using the Human Protein Atlas database, and the differential expression of TOP2A was verified by immunohistochemistry. Then, SRTING database and Cytoscape were used to establish PPI network for TOP2A and protein–protein interaction analysis was performed. The Oncomine database and cBioPortal were used to express and identify TOP2A mutation-related analyses. The expression differences of TOP2A gene were identified by LinkedOmics, and the GO and KEGG pathways were analyzed in combination with related genes. Finally, Kaplan–Meier survival analysis was performed to analyze the clinical and prognosis of HCC patients.

**Results:**

TOP2A may be a new biomarker and therapeutic target for hepatocellular carcinoma.

**Supplementary Information:**

The online version contains supplementary material available at 10.1186/s12935-021-02439-0.

## Introduction

Hepatocellular carcinoma (HCC) is a malignant form of primary liver cancer. According to 2020 world Health Organization statistics, HCC ranks sixth in incidence and fourth in mortality [1]. the incidence rate of HCC is obvious, and most of the cases come from less developed areas. Among them, Asia Pacific and sub-Saharan Africa are the highest incidence rate of HCC in the world [2]. the pathogenesis of HCC is complex. According to the research, HBV, HCV, aflatoxin, obesity, metabolic syndrome, and genetic factors are the risk factors of HCC [3]. At present, the common clinical diagnostic methods of HCC are ultrasound (US) and serum alpha fetoprotein (AFP), but the diagnostic results lack specificity and sensitivity [4]. In order to better diagnose HCC at an early stage, new biomarkers have become a research hotspot in recent years [5]. in recent ten years, although the treatment of HCC has been improved [6], the five-year survival rate is still not ideal [7]. At present, the therapeutic drugs for HCC have high cytotoxicity and adverse side effects. Therefore, there is an urgent need for new low toxicity drugs that can effectively treat HCC [8].

DNA topoisomerase is an enzyme that can control and change the topological state of DNA in the process of transcription. It can be divided into two types according to the short single strand or double strand breaks in DNA, type I and type II [9]. DNA topoisomerase 2-α (TOP2A) gene is located on human chromosome 17 (17q21-22) and encodes DNA topoisomerase II α [10]. DNA topoisomerase II α can control and change the topology of DNA, chromosome separation and cell cycle process [11]. In recent years, more and more research has shown that TOP2A significantly had expressed in tumor tissues (P < 0.001), and negatively correlated with the prognosis of patients with tumor (P = 0.002). Many studies have shown that TOP2A can be a prognostic biomarker and potential therapeutic target for bladder cancer, such as bladder urothelial carcinoma (BLCA), lung adenocarcinoma, prostate cancer, colon cancer and breast cancer [12–16]. Studies have found that TOP2A mRNA and TOP2A protein show significant levels in HCC, indicating that TOP2A is overexpressed in HCC [17], indicating that TOP2A may also be a potential biomarker of HCC. Many TOP2A inhibitors, such as anthracycline drugs (doxorubicin, epirubicin) and epipodophyllotoxin (etoposide, tiniposide) have been widely used in clinic [18]. However, the traditional TOP2A inhibitors have serious side effects and the therapeutic effect is not ideal. Therefore, it is urgent to find new TOP2A inhibitors with less side effects [19].

However, from the existing literature, there are relatively few reports on the mechanism of TOP2A in HCC. Therefore, we hope to study the mechanism of TOP2A in HCC through bioinformatics analysis. To provide new scientific basis and treatment methods for further study of the pathogenesis of liver cancer and biological treatment of tumor.

## Materials and methods

### GEO database

The NCBI-Gene Expression Omnibus (GEO) database (http://www.ebi.ac.uk/arrayexpress/) is an international public gene data repository. The database is managed by the National Center for Biotechnology Information of the National Library of Medicine. Center for Biotechnology Information, NCBI) established and performed data maintenance, used by international research institutions and groups to submit high-throughput microarrays and next-generation sequence functional genomics data sets for detection and analysis. GEO data information includes genes, Proteomic analysis, non-coding RNA analysis, etc., the data contained in it represents the original research results deposited by international research institutions. In this study, two data sets related to hepatocellular carcinoma (LIHC), GSE87630 and GSE45267, were selected and used by screening data sets, and a totally of 1995 different genes were obtained.

### Hub gene selection and survival and expression analysis.

The Wien analysis was performed through the two downloaded data sets, and the analysis results obtained were used to select the Hub gene through the MCC algorithm in the Cytoscape cytoHubba application. The Kaplan–Meier database (https://kmplot.com/analysis/) was used for the overall survival analysis of the hub gene, and genes that were not statistically significant were removed (P > 0.05). At the same time, use the GEPIA (Gene Expression Profiling Interactive Analysis) database to verify the contained DEGs, and finally leave the highly expressed and meaningful genes.

### TIMER database analysis

The Tumor Immune Estimation Resource (TIMER) database contains 32 cancers and 10,897 tissue sample information from the TCGA database. It can systematically analyze the correlation between one or more tumors and immune cell infiltration, and related genes in tumors. Correlation between expression in tissues and prognosis, gene mutations and copy number of cancer patients. In this study, using the TIMER database, input "GENE SYMBLE: TOP2A" to verify the expression and prognosis of this gene in different types of cancers, and analyze including B cells, CD4 + T cells, CD8 + T cells, and neutrophils. The prognosis of immune cell infiltration of cells, macrophages, and dendritic cells in patients with lung adenocarcinoma, the infiltration in the tissues and the correlation with TOP2A. Analyze the correlation between TOP2A and the surface markers of immune cells through the "Gene" module.

### Human Protein Atlas database analysis

According to different dimensions, the HPA (https://www.proteinatlas.org/) database is divided into three sections: Cell, Tissue and Pathology, which respectively show the expression of proteins in cells, normal tissues, and cancerous tissues. Filter according to the needs of the database. Search for the gene "TOP2A" in the search box, use the HPA database to analyze the expression of TOP2A mRNA and its antibodies HPA006458, HPA026773 and CAB002448 in normal liver tissues and liver cancer tissues, and then use immunohistochemistry experiments to verify that TOP2A is in normal tissues and liver cancer tissues expression.

### Immunohistochemical experiment

Paraffin specimens of 30 cases of hepatocellular carcinoma were collected from the Pathology Department of the First Affiliated Hospital of Guangxi Medical University. 30 cases of liver cancer tissue samples and tissue adjacent to carcinoma specimens by immunohistochemical staining. Immunohistochemistry (IHC) was used to assess THE expression of TOP2A in liver cancer and adjacent tissues. All samples to be measured were embedded in paraffin and fixed, and sectioned consecutively for use. Routine immunohistochemical staining was performed after HE staining. Each step strictly follows the SP kit operation instructions. Immunohistochemical results showed that TOP2A was expressed as brown particles under the microscope. Cell staining distribution was observed at low power (200x) to observe cell morphology and staining position. Five event horizons were randomly selected and recorded. In this study, Image J software was used for quantitative analysis and comparative analysis of the proportion of tumor lesions in 30 patients with liver cancer. Immunohistochemical images were intuitively quantitatively analyzed to understand the difference between liver cancer lesions and adjacent tissues in the progression of liver cancer. The higher the mean level, the stronger the optical density, the stronger the positive expression.

### Protein interaction PPI network construction and module analysis

The SRTING Database (https://cn.string-db.org/) is an online search for known protein mutual-aid relationships. Search conditions: (1) Protein: TOP2A; (2) Homo sapiens. Next, after downloading the data from the SRTING database, use Cytoscape, a software focused on open-source network visualization and analysis. Its core is to provide basic functional layout and query network, and build protein mutual PPI network based on the combination of basic data into a visual network. And the plug-in Molecular Complex assay (MCODE) for Cytoscape (version 1.4.2) identifies the paramount modules in the PPI network (MCODE score > 5-degree cutoff = 2, maximum depth = 100, K-core = 2, node cutoff = 0.2).

### c-BioPortal database analysis

c-BioPortal (http://cbioportal.org) is an open-access resource for exploring, visualizing and analyzing multi-dimensional cancer genome data. It currently contains 225 cancer studies. We used c-BioPortal to analyze the changes of TOP2A gene mutations in TCGA LIHC samples.

### Oncomine database analysis

The mRNA expression of TFF1 in ESCA was analyzed in the Oncomine 4.5 database. Oncomine (www.oncomine.org) is currently the world's largest oncogene chip database and comprehensive data mining platform, containing 715 gene expression data sets and data from 86,733 cancer tissues and normal tissues. The screening conditions of this study: "Gene: TOP2A"; "Cancer Type: Liver cancer"; "Analysis Type: Cancer vs. Normal Analysis"; "Data Type: mRNA". Select the box plot to display the results.

### LinkedOmics database analysis

The Linked Omics database (http://www.linkes.org/login.php) is a web-based platform for analyzing 32 TCGA cancer-related cubes. The Link Finder module of Linked Omics was used to study the differentially expressed genes related to TOP2A in the TCGA HCC cohort, and the Pearson correlation coefficient was used for statistical analysis. All results are presented graphically in a volcano map, heat map or scatter plot. The Link-Interpreter module of Linked Omics performs pathway and network analysis of differentially expressed genes. Use the comprehensive functional classification database in the Web-based Web Gestalt to sign and sort the data in the Link Finder results, and use GSEA to analyze the GO (CC, BP, MF) and KEGG channels.

### BioGPS database analysis

Use BioGPS (http://biogps.org/) database analysis: TOP2A gene expression in normal human tissues, enter "TOP2A" in the BioGPS database search box to search, select Human as the species.

### Extracting data from Kaplan Meier-plotter database

The Kaplan Meier-Plotter database (http://kmplot.com/analysis/) is a database that analyzes the clinical and prognosis of patients based on a variety of mRNA expression levels. The screening method of this study: ① "Cancer: Liver cancer"; ② "Gene: TOP2A (7153)"; ③ "Survival: OS (overall survival)"; ④ "Survival: RFS (recurrence-free survival, recurrence-free survival)"; ⑤ "Survival: PFS (progression-free survival, progression-free survival); ⑥ "Survival: DSS (disease-specific survival, disease-specific survival) ".

## Results

### Differentially expressed genes were obtained by screening data sets

**Fig. 1:**
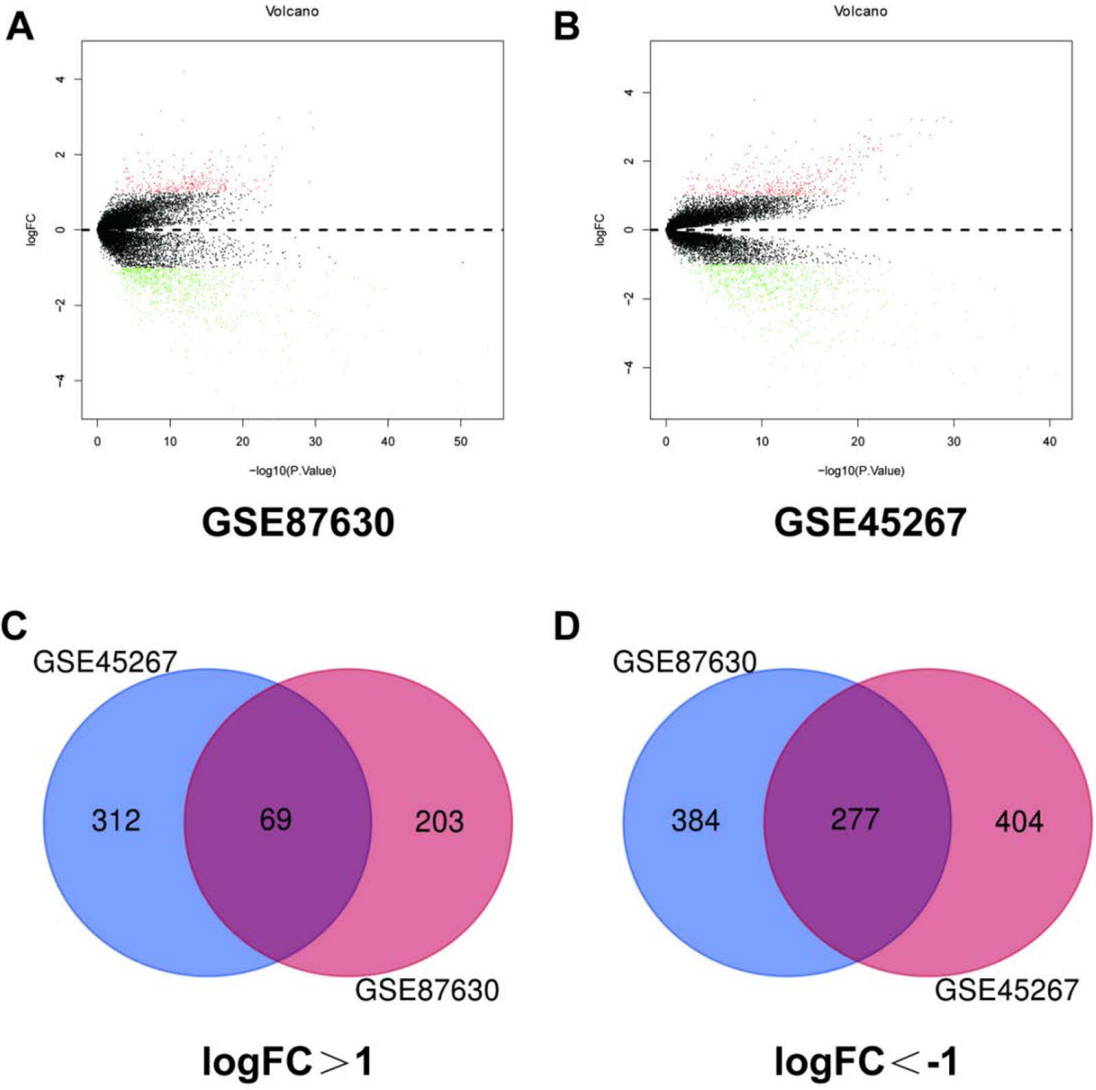
Differential expression of multiple genes in the two sample data sets. **A** GSE87630 data, **B** GSE45267 data. Identification of significantly differentially expressed genes in HCC **C** GSE87630 and GSE45267 data established the intersection of DEGs **D** GSE87630 and GSE45267 data established the intersection of DEGs. Taking |logFc|> 1 as the boundary value, P < 0.05

Through screening NCBI Gene Expression Omnibus GEO (https://www.ncbi.nlm.nih.gov/geo/) database GSE87630 and GSE45267, 2 groups of HCC-related data sets were screened out, a total of 181 samples, of which 71 liver cancer tissues There are 110 normal samples. Figure 1A, B shows the differential expression of multiple genes in the two sample data sets. By screening the GSE87630 and GSE45267 data sets (corrected p value < 0.05, |logFc|> 1), a total of 1995 differentially expressed genes DEGs were obtained. Use Venn diagram software to analyze common differentially expressed genes in the data set. A total of 346 deg were detected, of which 69 were up-regulated (Fig. 1A) and 277 were down-regulated (Fig. 1B).

### Construct a PPI network diagram of differentially expressed genes

**Fig. 2:**
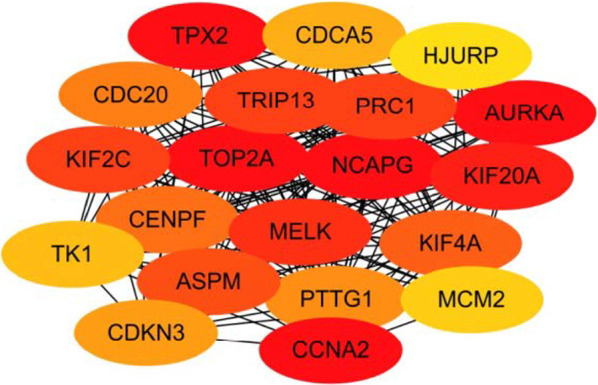
Identification of hub genes in the protein interaction (PPI) network. Cytoscape's plug-in cytoHubba uses the MCC algorithm to select the most important module from the PPI network

Use STRING to construct the PPI network diagram of DEGs, and use Cytoscape's plug-in cytoHubba to identify the most important nodes using the MCC algorithm, and finally extract 20 central nodes. (Fig. 2) The darker colors in the picture are the hub genes: TPX2, KIF2C, TOP2A, NCAPG, AURKA, KIF20A, CCNA2. These seven genes may serve as potential prognostic markers and therapeutic targets for liver cancer.

### Assess the prognostic value of the hub gene

**Fig. 3:**
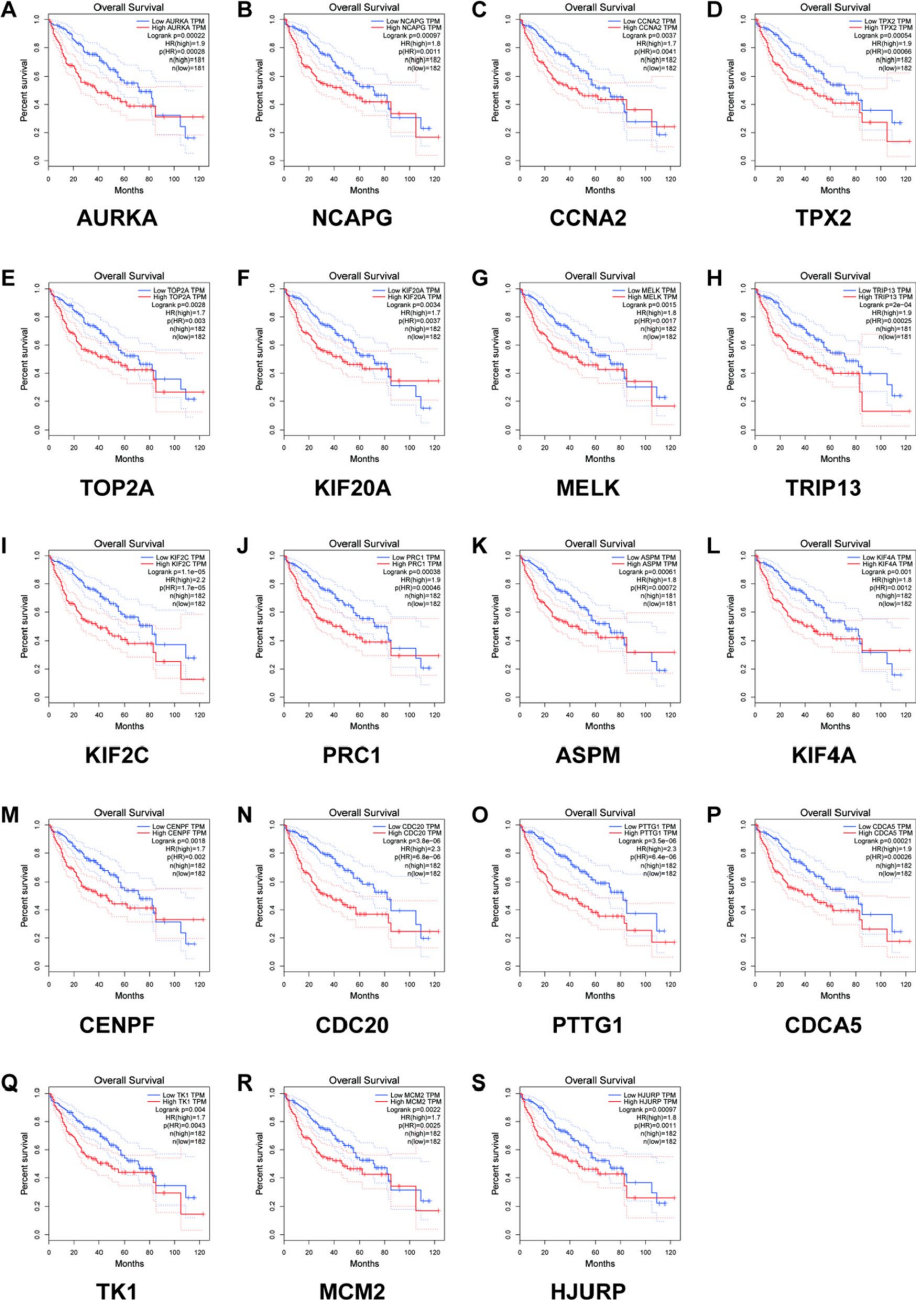
Prognostic analysis of 20 key genes (AURKA (**A**), NCAPG (**B**), CCNA2 (**C**), TPX2 (**D**), TOP2A (**E**), KIF20A (**F**), MELK (**G**), TRIP13 (**H**), KIF2C (**I**), PRC1 (**J**), ASPM (**K**), TKIF4A (**L**), CENPF (**M**), CDC20 (**N**), PTTG1 (**O**), CDCA5 (**P**), TK1 (**Q**), MCM2 (**R**), HJURP (**S**)

We use STRING to construct the PPI network diagram of DEGs. Cytotype MCODE was used to identify the most important nodes, and finally 20 central nodes were extracted to obtain 20 hub genes, and the prognostic value of these 20 genes was analyzed and evaluated in the GEPIA database (P value < 0.05), and no statistical significance was removed. Generally speaking, the greater the distance between the two curves (the greater the branch), the greater the difference in prognosis (end-point event rate) between the two groups. Using this as a standard, 19 genes AURKA, NCAPG, CCNA2, TPX2, TOP2A, KIF20A, MELK, TRIP13, KIF2C, PRC1, ASPM, TKIF4A, CENPF, CDC20, PTTG1, CDCA5, TK1, MCM2, HJURP were finally obtained. (Fig. 3).

### Verify the expression level of core genes in HCC patients and normal people

**Fig 4:**
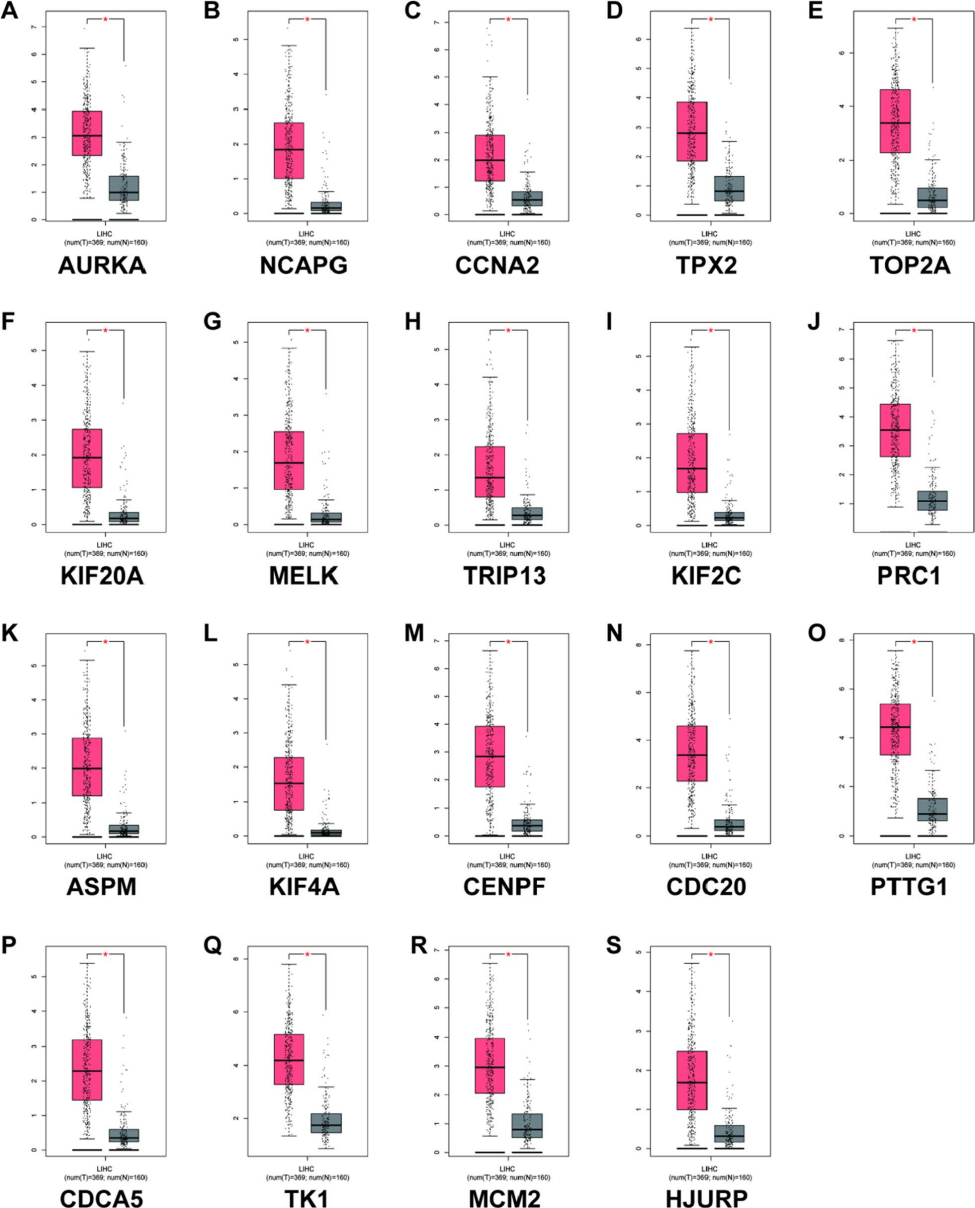
26 key genes (AURKA (**A**), NCAPG (**B**), CCNA2 (**C**), TPX2 (**D**), TOP2A (**E**), KIF20A (**F**), MELK (**G**), TRIP13 (**H**), KIF2C (**I**), PRC1 (**J**), ASPM (**K**), TKIF4A (**L**), CENPF (**M**), CDC20 (**N**), PTTG1 (**O**), CDCA5 (**P**), TK1 (**Q**), MCM2 (**R**), HJURP (**S**) Expression level. "*" means "P value < 0.05". The y-axis represents the relative expression value log2 (TPM + 1)

Use the GEPIA database to verify 19 extracted core genes (KIF2C, CDC20, TPX2, TK1, CDKN3, CENPF, TOP2A, TRIP13, CDCA5, ASPM, MELK, NCAPG, PRC1, HJURP, AUPKA, KF20A, KIF4A, PTTG1, CCNA2, MCM2) Expression levels in HCC patients and normal subjects. Compared with normal liver samples, most of the extracted genes are highly expressed in HCC tissue samples. Among the 19 candidate genes, 19 genes AURKA, NCAPG, CCNA2, TPX2, TOP2A, KIF20A, MELK, TRIP13, KIF2C, PRC1, ASPM, TKIF4A, CENPF, CDC20, PTTG1, CDCA5, TK1, MCM2, HJURP are high in HCC Express. (Fig. 4).

### TOP2A expression in cancer tissues

**Fig. 5:**
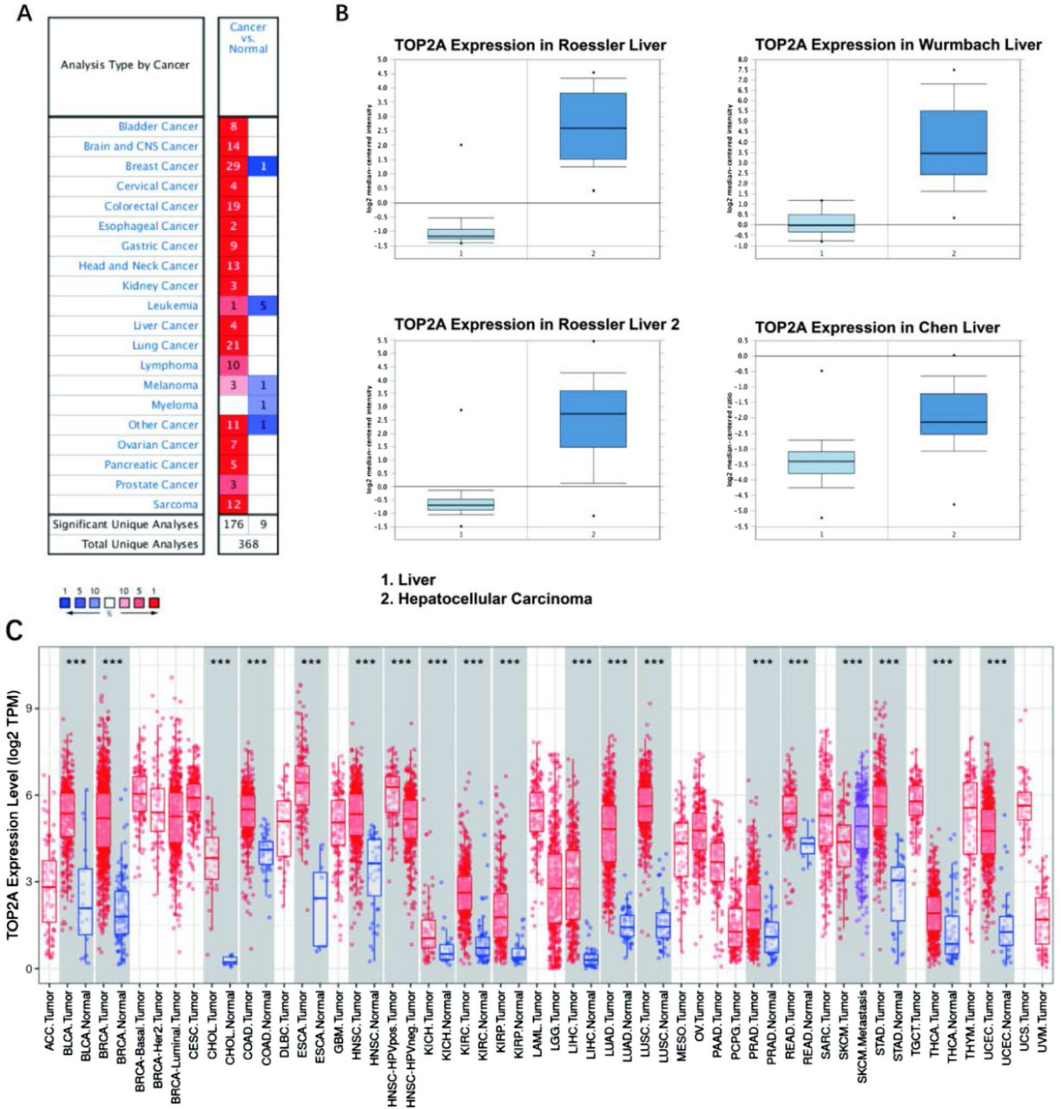
**A** The expression of TOP2A in different tumors in the Oncomine database. **B** Differential expression of TOP2A in cancer tissues and normal tissues. **C**The level of expression of TOP2A in all tumor tissues and cancer-side tissues (*P < 0.05 **P < 0.01; ***P < 0. 001)

First, analyze the mRNA expression of TOP2A in different tumor tissues. The Oncomine database has important functions such as gene expression differences, gene expression and clinical correlation, and multi-gene co-expression analysis. The Oncomine database was used to detect the expression of TOP2AmRNA in different tumors and normal clinical specimens. In the end, 693 data sets including 83,857 samples were selected. Compared with normal tissues, TOP2A mRNA is significantly expressed in bladder cancer, head and central nervous system cancer, breast cancer, cervical cancer, colorectal cancer, and other cancers. Up-regulation, but down-regulation in a small number of cancers such as head and central nervous system cancer, Leukemia, malignant melanoma, and myeloma (Fig. 5A, B). Prompt TOP2A transcription level mainly depends on tumor type. According to the data in the database in Oncomine 4.5 TOP2A in HCC tissue than mRNA expression in normal tissue. The differential expression of TOP2A in cancer tissues and normal tissues was obtained from the TIMER database. The expression of TOP2A in cancer tissues such as kidney cancer, liver cancer, and breast cancer were significantly higher than that in normal tissues, and TOP2A gene expression was highest in hepatocellular carcinoma stage III (Additional file 1: Fig. S1). As shown in Table 1, there was no significant difference in gender, family history of cancer, residual tumor between the two groups (all P > 0.05).

**Table 1:** Association between TOP2A expression and the clinical parameters in patients with hepatocellular carcinoma in TCGA

	Total (N = 339) (%)	TOP2A Expression		P-value
		High (N = 113) (%)	Low (N = 226) (%)	
Age (year)				0.00399
< 65	208 (61.4)	82 (72.6)	126 (55.8)	
≥ 65	131 (38.6)	31 (27.4)	100 (44.2)	
Gender				0.108
Male	231 (68.1)	70 (61.9)	161 (71.2)	
Female	108 (31.9)	43 (38.1)	65 (28.8)	
Family history of cancer				0.148
No	196 (57.8)	72 (63.7)	124 (54.9)	
Yes	98 (28.9)	25 (22.1)	73 (32.3)	
Unknown	45 (13.3)	16 (14.2)	29 (12.8)	
TNM stage				< 0.001
I	170 (50.1)	36 (31.9)	134 (59.3)	
II	84 (24.8)	37 (32.7)	47 (20.8)	
III	81 (23.9)	40 (35.4)	41 (18.1)	
IV	4 (1.2)	0 (0)	4 (1.8)	
Histologic grade				< 0.001
G1–G2	212 (62.5)	52 (46.0)	160 (70.8)	
G3–G4	125 (36.9)	60 (53.1)	65 (28.8)	
Unknown	2 (0.6)	1 (0.9)	1 (0.4)	
Ishak score				0.00957
0–4	124 (36.6)	34 (30.1)	90 (39.8)	
5–6	74 (21.8)	19 (16.8)	55 (24.3)	
Unknown	141 (41.6)	60 (53.1)	81 (35.8)	
Child–Pugh grade				0.0119
A	207 (61.1)	59 (52.2)	148 (65.5)	
B-C	21 (6.2)	5 (4.4)	16 (7.1)	
Unknown	111 (32.7)	49 (43.4)	62 (27.4)	
Vascular invasion				0.0205
None	193 (56.9)	53 (46.9)	140 (61.9)	
Micro	84 (24.8)	30 (26.5)	54 (23.9)	
Macro	14 (4.1)	6 (5.3)	8 (3.5)	
Unknown	48 (14.2)	24 (21.2)	24 (10.6)	
Alpha fetoprotein				< 0.001
Negative	143 (42.2)	25 (22.1)	118 (52.2)	
Positive	120 (35.4)	54 (47.8)	66 (29.2)	
Unknown	76 (22.4)	34 (30.1)	42 (18.6)	
Residual tumor				0.511
R0	301 (88.8)	99 (87.6)	202 (89.4)	
R1-R2	12 (3.5)	3 (2.7)	9 (4.0)	
Unknown	26 (7.7)	11 (9.7)	15 (6.6)	
Living status				< 0.001
Alive	224 (66.1)	60 (53.1)	164 (72.6)	
Dead	115 (33.9)	53 (46.9)	62 (27.4)	
Disease status				0.00479
No	163 (48.1)	41 (36.3)	122 (54.0)	
Yes	132 (38.9)	57 (50.4)	75 (33.2)	
Unknown	44 (13.0)	15 (13.3)	29 (12.8)	

In this study, the differential expression of TOP2A in tumor tissues and normal tissues was also analyzed online from the TIMER database. The results showed that TOP2A was differentially expressed in a variety of tumors, and the expression of TOP2A was higher in hepatocellular carcinoma tissues than in adjacent tissues (p < 0.001) (Fig. 5C).

Online analysis of the BioGPS database shows that TOP2A gene is expressed in most normal tissues of the human body, while the expression level in liver tissue is slightly higher than the average value of 10.2, and the median value of expression is 16.65 (Additional file 2: Fig. S2).

From the results of these databases, it can be concluded that TOP2A is highly expressed in cancer tissues and low expressed in normal tissues. We used these databases to verify the expression of TOP2A with each other. Since the original data sources of these databases were different, we used the data from different sources to verify each other, which increased the truthfulness and credibility of the conclusion.

### The expression of TOP2A in HCC

**Fig. 6:**
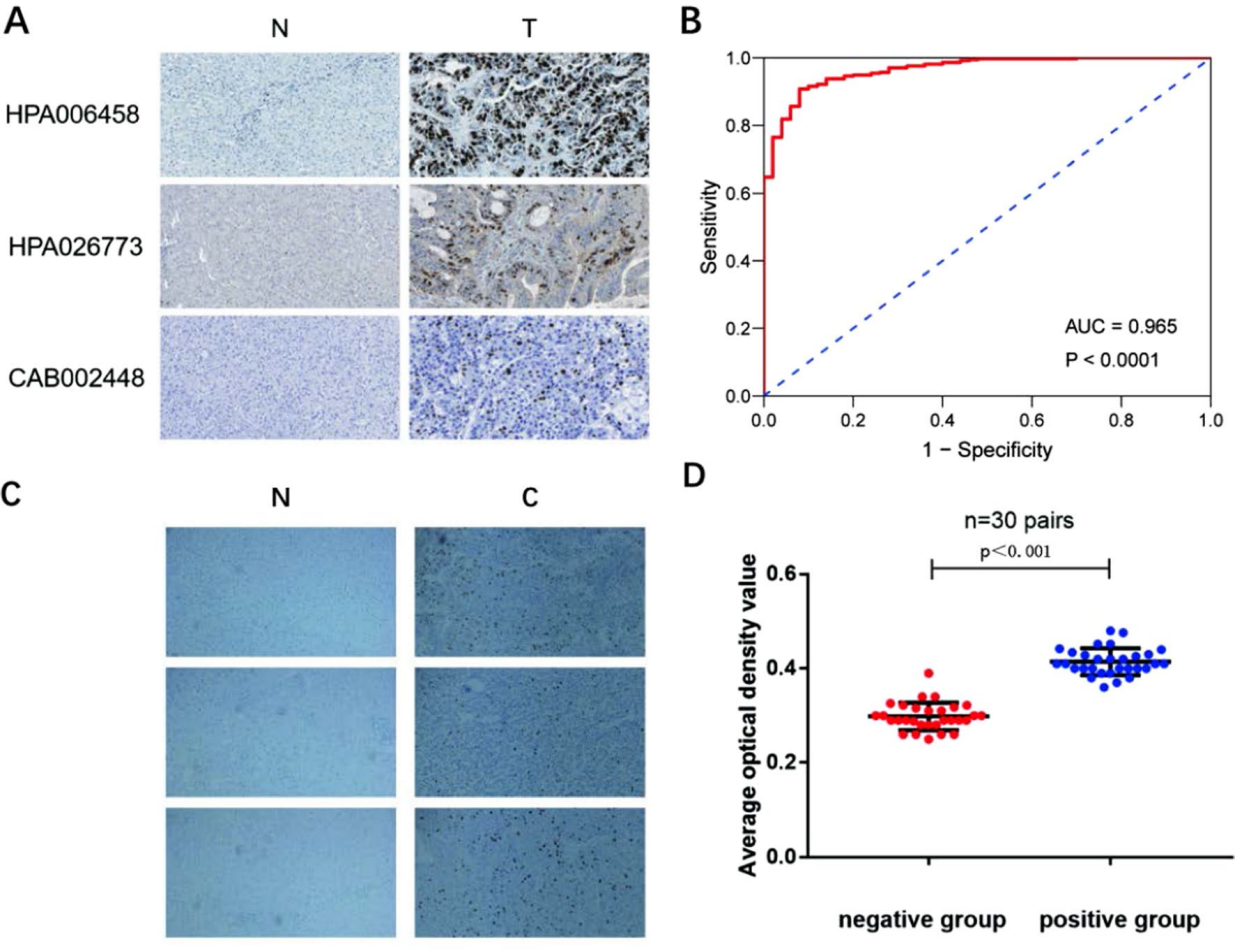
Partial immunohistochemical results of TOP2A gene in normal human liver tissue and patient liver cancer tissue. **A** Immunohistochemical results of three antibodies related to TOP2A in normal tissues and liver cancer tissues. **B** Validation of diagnostic value of TOP2A upregulation for HCC using ROC curve. **C** Part of the results of immunohistochemistry experiments. **D** Scatter plot of average optical density of liver cancer tissues and adjacent tissues of 30 liver cancer patients

By analyzing the HPA database, the following results are obtained: The high expression of TOP2A in HCC is related to three antibodies, namely HPA006458, CAB002448, and HPA026773. In order to verify the expression of TOP2A in HCC, we selected the expression of these three antibodies in normal tissues and liver cancer tissues. The results showed that in normal tissues, their expression levels are low or even not expressed. In cancer cells, their expressions are all Raised. Immunohistochemical staining results and analysis showed that TOP2A was localized in the nucleus. Antibody HPA006458, antibody CAB002448, and antibody HPA026773 are mainly expressed in the nucleoplasm, and their expression in HCC is significantly higher than that in normal tissues (Fig. 6A). The diagnostic value of TOP2A upregulation for HCC was also confirmed by ROC curves (AUC = 0.965, P < 0.0001) (Fig. 6B).

A total of 30 pairs of cancer tissue samples from 30 patients with liver cancer in the People's Hospital of Guangxi Zhuang Autonomous Region were collected, and immunohistochemical staining was performed. All the staining steps were performed in strict accordance with the standard protocol. Randomly select 5 fields of view from each sample, use Image J to determine the average optical density value, and use SPSS 19.0 (IBM Inc.) for statistical analysis.

With cancer of the liver cancer tissue samples and normal tissue in patients with five horizons of the average value of the average optical density do scatterplot, cancer tissue samples of the average optical density value were higher than the average optical density value of the normal tissue (Fig. 6C, D). The results observed by immunohistochemistry are consistent with the results we obtained in the HPA database, indicating that TOP2A can be used as a biomarker and potential prognostic value for liver cancer.

### The establishment and analysis of PPI network

**Fig. 7:**
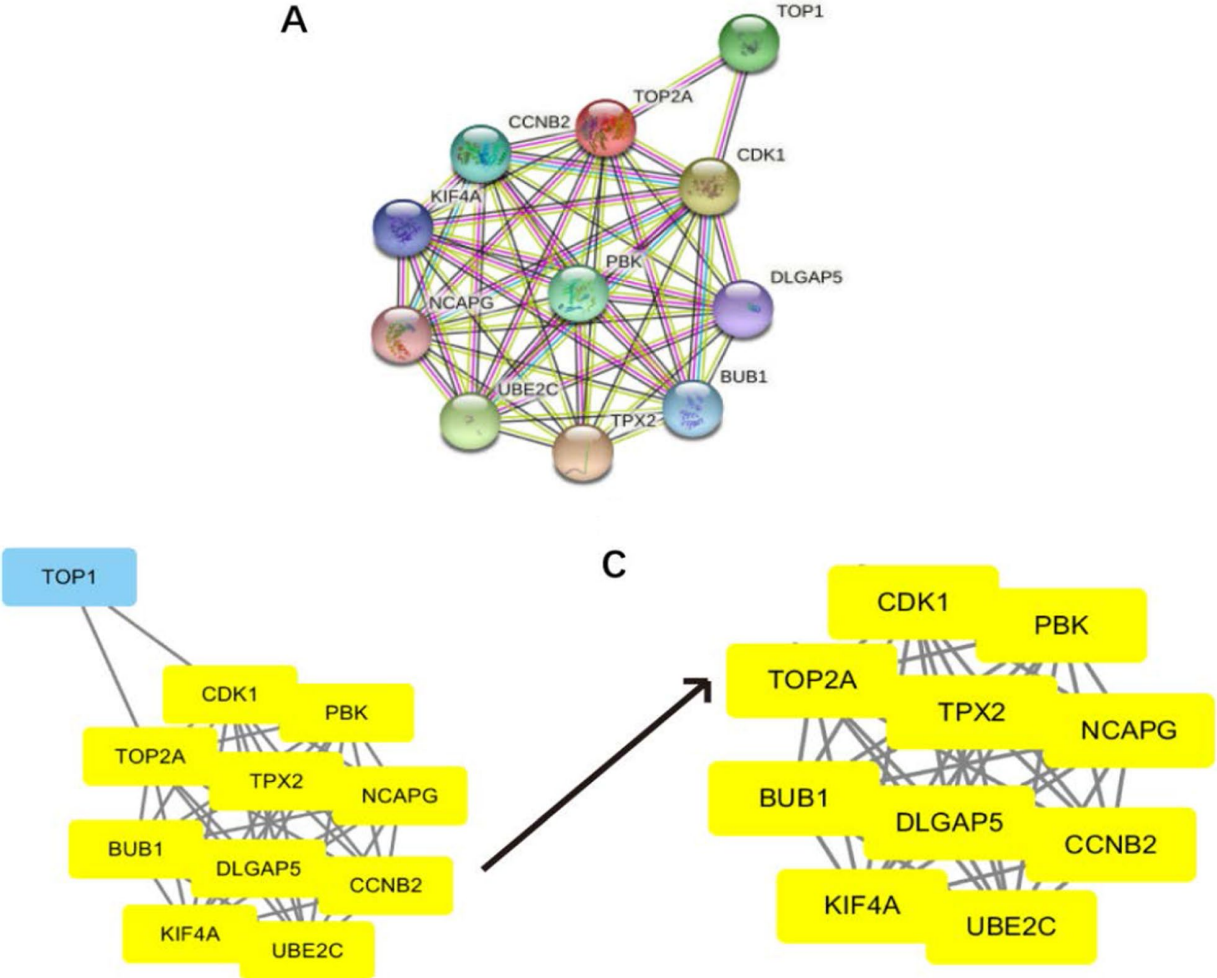
PPI network diagram and the hub gene identification **A** STRING Protein Mutual Network **B** uses Cytosacape to build the most important module of the PPI network **C** selected from the PPI network using Cytocsape's plug-in MOOD

In the STRING database, complex network diagrams represent data on protein interactions, where nodes correspond to proteins and edges represent interactions between proteins. When part of the network graph formed in the STRING database overlaps, it can be characterized as a pathway or a functional graph. Even antagonistic proteins can be functionally related, like activators and inhibitors within a pathway. Construct the PPI network diagram of DEG using STRING (Fig. 7A). Cytoscape software was used to analyze the PPI network formed by each gene, and the top 10 genes, CDK1, PBK, TOP2A, TPX2, NCAPG, BUB1, DLGAP5, CCNB2, KIF4A and UBE2C, which were most associated with other nodes were counted (Fig. 7B, C). Their findings are helpful to reveal the pathogenesis of HCC and to search for therapeutic targets and prognostic biomarkers.

### Genomic mutation of TOP2A in HCC

**Fig. 8:**
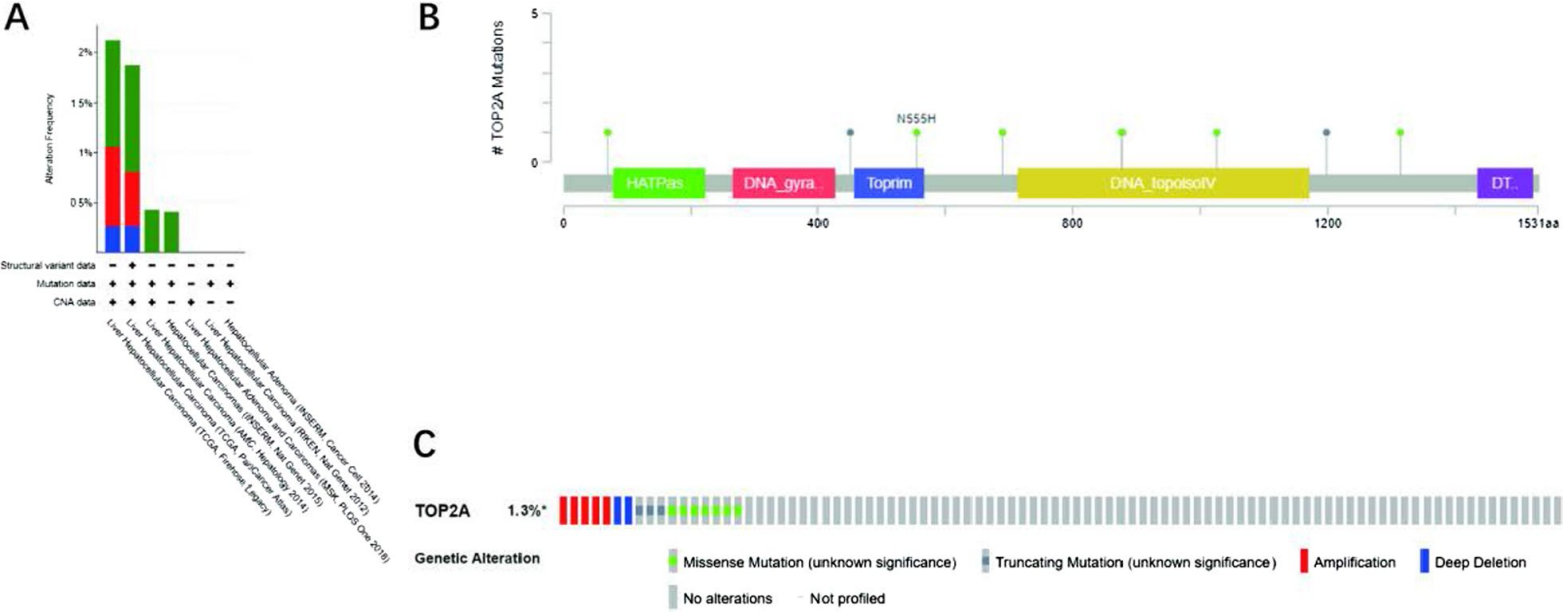
**A** Mutation sites for mutations in the mutation frequency **B** TOP2A gene expression of the TOP2A gene in different data sets **C** Type and frequency of TOP2A gene mutations in LIHC

We analyzed the gene mutations of TOP2A and CNAs in the samples of HCA in 8 databases using the cBioPortal database, and the results showed that there were 8 mutation sites in the TOP2A gene (Fig. 8B), and that TOP2A had different mutation frequencies in different data sets, 2.12% (TCGA, Firehose Legacy), 1.88% (TCGA, PanCance Atlas), and 0.43% AMC, Hepatology 2014), 0.41% (INSERM, Nat Genet 2015), TCGA data analysis shows that HCA patients have the largest number of TOP2A CNAs, mainly due to genetic mutations, about 0.5%-1% (Fig. 8A). The study also analyzed HCA data from 442 TCGA cases, PanCancer Altas, and 372 cases of TCGA, Firehose Legacy, and showed that liver cancer CNAs were closely related to top2A expression levels (single-factor variance analysis, p < 0.05) (Fig. 8C). Secondly, we use cBioPortal to determine the type and frequency of TOP2A changes in LIHC based on the sequencing data of LIHC patients in the TCGA database. Of the 1297 LIHC patients, 17 had a change in TOP2A. These changes were missing 2 (0.15%) and 5 (0.39%) respectively, so amplification is the most common type of TOP2A mutation in LIHC.

### Analysis of co-expression genes associated with TOP2A in HCC

**Fig. 9:**
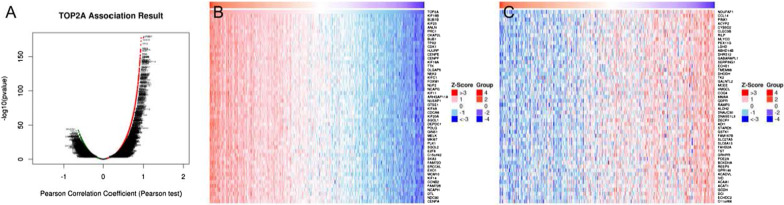
The co-expression analysis results of TOP2A. **A** The mRNA sequencing data **B** positively correlated gene **C** TOP2A of the MRNA sequencing data **B** of HCC patients

The mRNA sequences of 371 patients with TCGA liver cancer were analyzed by functional module method。As can be seen from the volcanic map, the number of genes with TOP2A showing significant positive correlation is higher than the number of genes with TOP2A showing significant negative correlation. Figure 9B, C shows 50 important genes that are positively correlated and negatively correlated with TOP2A. These two results show that TOP2A shows strong positive expression with KIF18B (drive protein family member 18B, Pearson correlation coefficient = 0.94, p s = 3.793e−178), BUB1B (Serine protein kinase B, Pearson Correlation coefficient = 0.93, p = 1.681e−173), KIF23 (Drive protein family member 23, Pearson correlation coefficient = 0.93, p = 4.906e−168) It is strongly correlated and reflects that TOP2A can participate in tumor development by driving proteins, serine protein kinases, etc.

### Analysis of co-expressed gene GO and KEGG pathways associated with TOP2A in HCC

The GO analysis of gene enrichment analysis (STRING) shows that the difference expression genes associated with TOP2A are mainly located in nucleoplasm, cytosols, nucleosomes, and cytoplasm, and are mainly involved in physiological processes such as cell division, mitochondrial division, G2/M transition of mitochondrial cell cycle, and cell proliferation. Their molecular functions include protein binding, ATP binding, protein kinase activity and protein serotonin/kinase activity. KEGG path analysis shows that TOP2A may play a role in HCC by participating in progesterone-mediated oocyte maturation pathways, oocyte subtraction division pathways, cell cycle pathways, and p53 signal transduction pathways.

### The prognosis of TOP2A in liver cancer

**Fig. 10:**
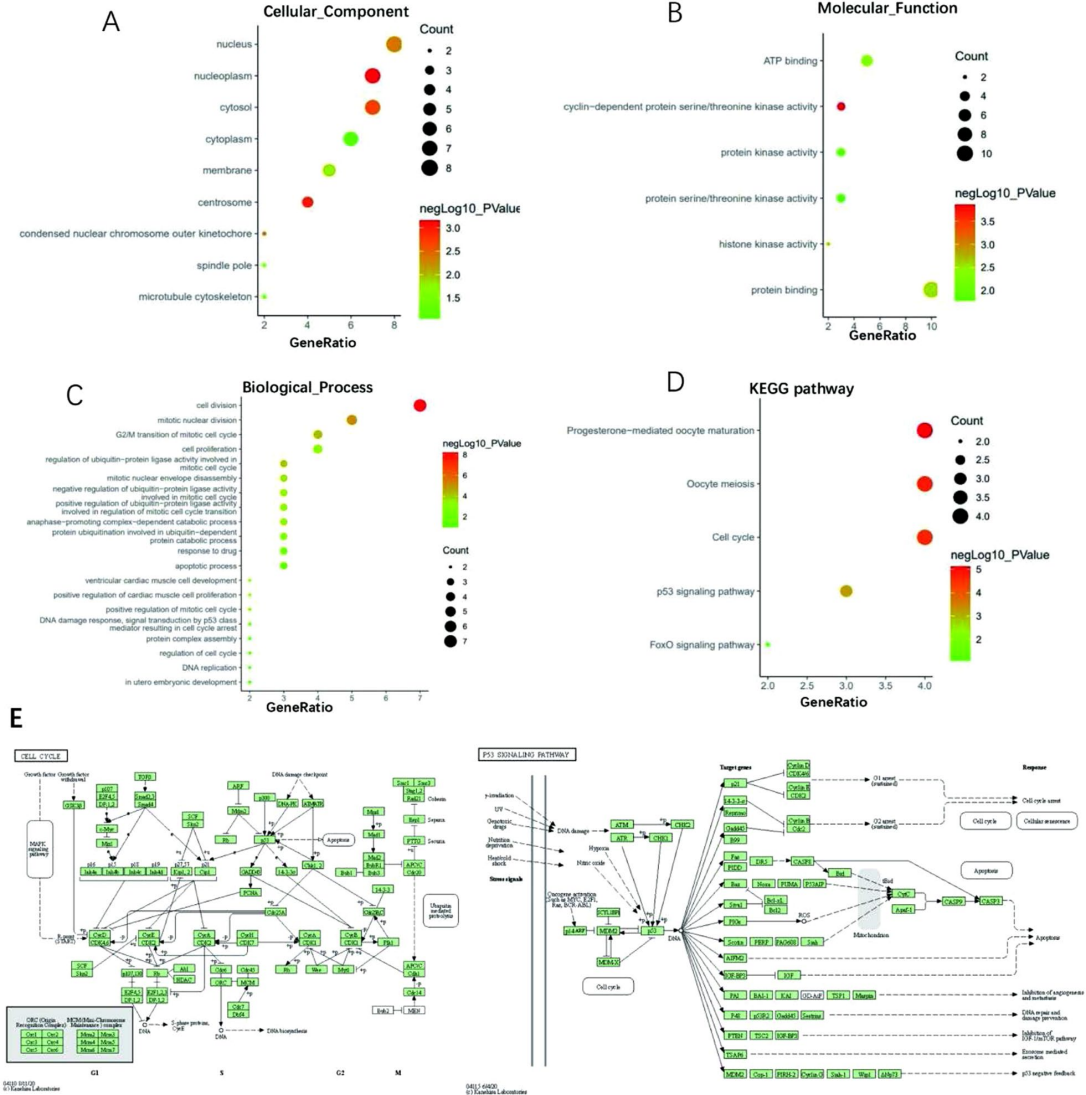
Notes on the GO functions of important DEGs and KEGG links. **A** The top 9 enriched cellular component (CC) items for the co-expressed genes of TOP2A. **B** The top 6 enriched molecular function (MF) items for the co-expressed items for the co-expressed genes of TOP2A. **C** The top 25 enriched biological process (BP) items for the co-expressed genes of TOP2A. **D** The top 5 enriched KEGG items for the co-expressed items for the co-expressed genes of TOP2A. **E** KEGG path notes for cell cycle pathways ang KEGG path notes for P53 signal paths

**Fig. 11:**
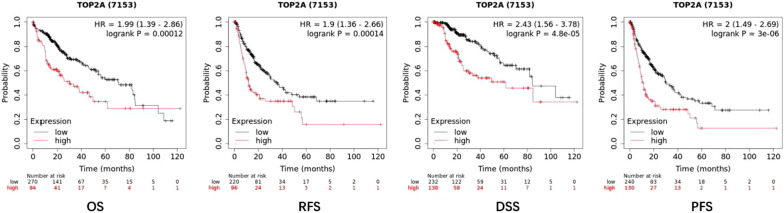
The expression of TOP2A's relationship with the prognosis of patients with LIHC

Analysis of TOP2A prognosis in the liver cancer database using the Kaplan–Meier Curve (www.kaplot.com) database website. Online survival analysis results (Fig. 10) show that the diagram below high TOP2A genes in liver cancer cells express the survival time of less than its survival rate in lower expression of liver cancer. Therefore, it can be shown that the survival rate of liver cancer patients is affected by the expression of TOP2A. By analyzing OS (Overall survival), RFS (Recurrence-free survival) and DSS (disease-specific survival), And PFS (progression—free survival), has obtained the high TOP2A gene expression patients survival time under low TOP2A gene expression in patients with as a result, it can explain TOP2A expression of genes that affect the prognosis of patients with liver cancer (Fig. 11). We performed the Cox regression analyses to explore the independent indicators of OS in HCC. In the univariate model, vascular invasion, TNM stage, and PARPBP expression were significantly related to OS in HCC (all P < 0.05). The following multivariate analyses confirmed that TOP2A overexpression was an independent indicator of unfavorable OS (HR = 1.21(1.04, 1.39), P = 0.011) in HCC after adjusting other prognostic indicators (Tables 2).

**Table 2:** Cox proportional hazards regression model analysis of overall survival

Variables	Univariate analysis		Multivariate analysis	
	HR (95% CI)	*p*	HR (95% CI)	*p*
Age (≥ 65 vs. < 65)	1.23 (0.85,1.78)	0.273	–	–
Gender (Female vs. Male)	1.26 (0.87,1.84)	0.228	–	–
Family history of cancer (YES vs. NO)	1.14 (0.76,1.69)	0.530	–	–
TNM stage (II vs. I)	1.42 (0.87,2.32)	0.160	1.14 (0.6,2.15)	0.694
TNM stage (III vs. I)	2.72 (1.78,4.15)	** < 0.001**	2.03 (1.19,3.47)	**0.010**
TNM stage (IV vs. I)	5.44 (1.68,17.63)	**0.005**	6.91 (2.09,22.89)	**0.002**
Histologic grade (G3–G4 vs. G1–G2)	1.14 (0.78,1.67)	0.489	–	–
Ishak score (5–6 vs. 0–4)	0.87 (0.5,1.5)	0.612	–	–
Child–Pugh grade (B–C vs. A)	1.66 (0.82,3.36)	0.159	–	–
Vascular invasion (Micro vs. none)	1.16 (0.72,1.88)	0.539	1.07 (0.61,1.87)	0.824
Vascular invasion (Macro vs. none)	2.52 (1.14,5.58)	**0.023**	1.84 (0.8,4.24)	0.150
Alpha fetoprotein (positive vs. negative)	1.45 (0.92,2.28)	0.108	–	–
Residual tumor (R1–R2 vs. R0)	1.17 (0.43,3.2)	0.754	–	–
TOP2A (high vs. low)	1.26 (1.11,1.42)	** < 0.001**	1.21 (1.04,1.39)	**0.011**

Statistically signifcant *p* values are given in bold *p* < 0.05

*HR* hazard ratio; *CI* confdence interval

### THE relationship between TOP2A expression and hepatocellular carcinoma immune cell immersion

**Fig. 12:**
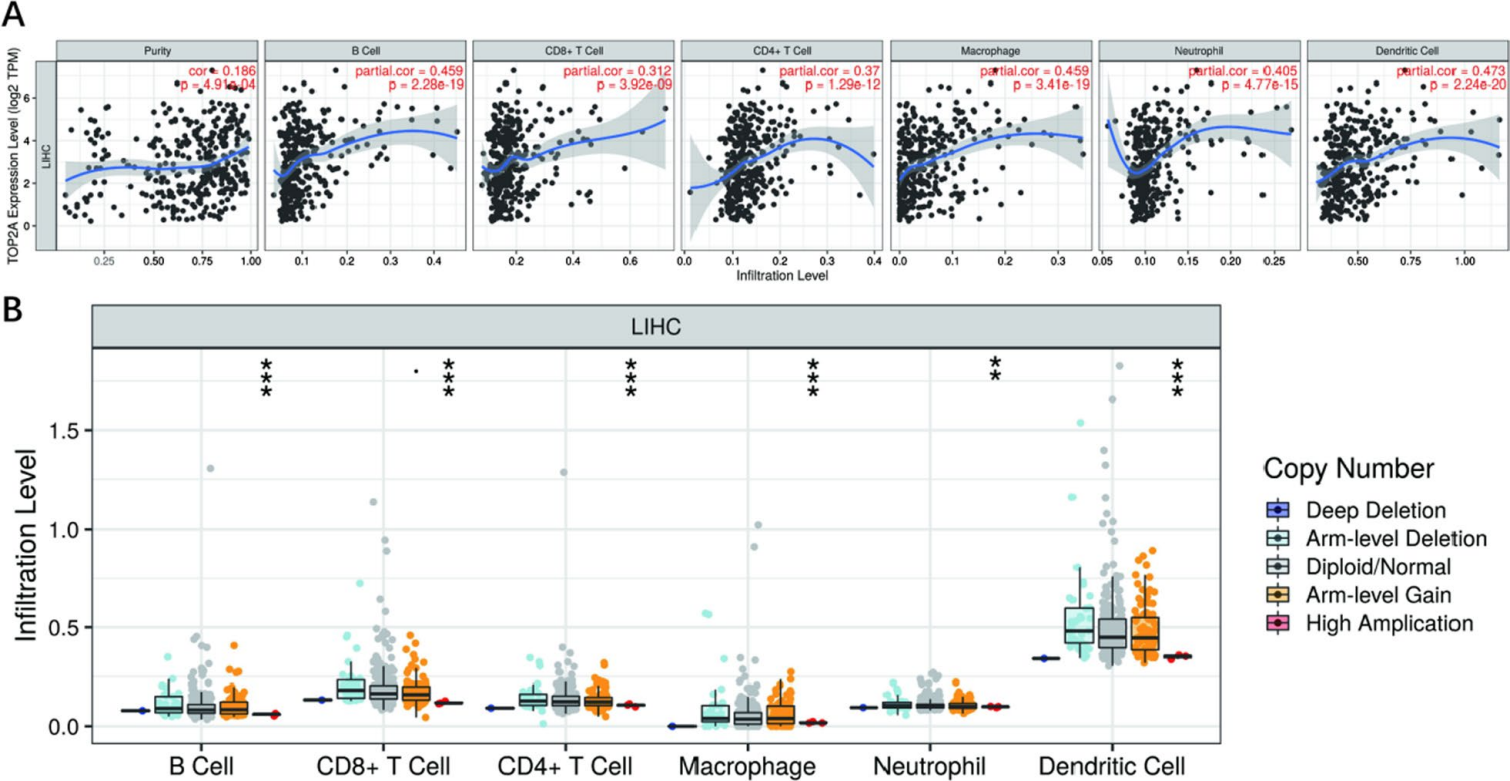
**A** Correlation between TOP2A expression level and hepatocellular carcinoma immune cell infiltration level. **B** Relationship between TOP2A expression and hepatocellular cancer tumor immune cell immersion. *P < 0.05, **P < 0.01; ***P < 0. 001

Using the TIMER database, the correlation between TOP2A expression and hepatocellular cancer immune cell immersion showed that TOP2A gene expression was related to the level of immune immersion of cell purity, B cells, CD8 plus T cells, neutrophils, macrophages, dendritic cells, which may be involved in the immune immersion process of liver cell cancer cells; The following illustration shows that TOP2A expression has some effect on immune infiltration and hepatocellular carcinoma cancer cell purity (r-0.186), which is positively correlated with B-cells (r-0.459) and CD8-T cells (r-0.312). There is a positive correlation with CD4-T cells (r-0.37), macrophages (r-0.459), neutrophils (r-0.405), and dendritic cells (r-0.473) (Fig. 12A). In addition, compared with normal tissue, different copy states of TOP2A have some effect on immune immersion (Fig. 12B).

## Discussion

Hepatocellular carcinoma (HCC) is a high mortality rate of primary liver cancer. Although hepatocellular carcinoma has made good progress in diagnosis and treatment, it has a high degree of malignancy, poor survival prognosis and a very low five-year survival rate. Therefore, exploring the detailed mechanisms of hepatocellular carcinoma pathogenesis and identifying promising diagnostic and prognostic biomarkers for hepatocellular carcinoma may help to provide effective therapeutic targets and improve patient outcomes [20]. Therefore, early analysis of HCC prognostic factors and screening of sensitive diagnostic markers is an important subject in HCC clinical research. The results of this study support the possibility that TOP2A may be considered a newly discovered biomarker for HCC diagnosis, prognosis, and treatment.

Based on a comprehensive expression analysis and survival analysis, 19 genes (ASPM, CCNA2, CDCA2, FAM83D, KIF2C, KIF20A, MCM2, MCM6, PRC1, TK1, TPX2, TROAP, AURKA, CDC20, CENPF, HJURP, KIF4A, MCM5, MELK, NCAPG, PTTG1, TOP2A, TRIP13 and UBE2T as a key gene that may be associated with HCC progression. Currently, many studies have shown that these core genes may act as carcinogenic genes or promising biomarkers of cancer. For example, Du et al. have identified AURKA as a target for cancer treatment, and have identified small molecule [21] that targets AURKA. Gong et al. research found that NCAPG plays a role as a cancer gene in HCC, promoting cell proliferation and anti-apoptosis by activating the PI3K/AKT/FOXO4 pathway [22]. Song et al. used pharmacogenomic analysis to show that CCNA2 is a predictive biomarker of polo-like kinase I inhibitor sensitivity in stomach cancer [23]. The research of Huang et al. shows that TPX2 silencing plays an anti-tumor role in hepatocellular carcinoma by regulating PI3K/AKT signaling pathway [24]. Jain et al. found overexpression of TOP2A as a therapeutic target for adrenal cortical carcinoma [25]. Shen et al. found that KIF20A can affect the prognosis of bladder cancer by promoting proliferation and metastasis of bladder cancer cells [26]. Liu et al. found that MELK accelerated the progression of colorectal cancer by activating the FAK/Src pathway [27]. Zhang et al. found that TRIP13 promotes cell proliferation, migration, and invasion of glioblastoma through the FBXW7/c-MYC axis [28]. Bai et al. determined that KIF2C was associated with the progression and prognosis of lung adenocarcinoma through co-expression network analysis [29]. Chen et al. found that the microtube-related protein PRC1 was associated with the Wnt/β-catenin signaling pathway to promote early recurrence of hepatocellular carcinoma [30]. Pai et al. found that the proportion of high ASPM-expressing cells in tumors was inversely proportional to the relapse-free survival rate of PCCA patients, and ASPM promoted the occurrence and progression of prostate cancer by enhancing Wnt-DVL-3 -β-catenin signaling [31]. Yang et al. found that KIF4A promoted proliferation of clear cell renal cell carcinoma (cRcc) in vitro and in vivo [32]. Sun et al. found that CENPF overexpression was associated with poor prognosis and tumor bone metastasis in breast cancer [33]. Li et al. found that increased CDC20 expression was associated with the occurrence and progression of hepatocellular carcinoma [34]. Huang, found that long chain noncoding RNA PTTG3P by raising PTTG1 and activating PI3K/AKT signaling pathway to promote cell growth and metastasis of liver cancer [35]. Tian et al. found that increased CDCA5 expression was associated with increased tumor diameter and microvascular invasion of HCC, and CDCA5 overexpression might be an indicator of poor prognosis in HCC patients, and its high expression was significantly associated with reduced survival rate [36]. Del Re et al. showed that Nuclear TK1 expression was an independent prognostic factor for survival of precancerous and malignant cervical lesions [37]. Research by Garbati et al. found that MCM2 and carbonate enzyme 9 are new potential targets for neuroblastoma therapy [38]. Studies by Chen et al. have shown that HJURP unstable p21 through MAPK/ERK1/2 and AKT/GSK3 beta signaling pathways, thereby promoting hepatocellular carcinoma proliferation [39].

The TOP2A gene, which is the subject of this study, is one of the genes that encodes DNA topological isomer enzymes by looking for literature and combining clinical results. DNA topoisomerases are enzymes that control the DNA topology in all cells and can be classified into two types, type I and type II [9], based on whether they produce transient single or double strand breaks in DNA. The topological isomer enzymes of eukaryotic cells are mainly divided into TOP1A and TOP2A. Among them, the intermediate product formed in the catalytic process causes the DNA double strand to break briefly called TOP2A. TOP2A plays an important role in important life processes such as cell DNA replication, transcription, and filamentation. In recent years, a growing number of studies have shown that TOP2A is significantly higher in tumor tissue (P < 0.001) and significantly negatively correlated with prognosis in tumor patients (P = 0.002).

Our results suggest that TOP2A is overexpressed in patients with hepatocellular carcinoma (HCC) and that its mRNA expression is significantly associated with individual cancer stage in HCC patients. Immunohistochemical experiments can also verify this view, and the top ten nodes associated with TOP2A were found in the protein mutual-aid network. In addition, the mutation rate of TOP2A in HCC patients was 1.3%, and amplification was the most common TOP2A mutation type in HCC, and mutations of TOP2A gene may also significantly affect the prognosis of HCC patients. Functional network analysis suggested that TOP2A may play a role in HCC by participating in progesterone mediated oocyte maturation pathway and oocyte meiosis pathway. The mechanisms that lead to the development of HCC are still largely unknown. This lack of knowledge is directly reflected in the identification and application of biomarkers for early diagnosis, which leads to poor treatment outcomes. Our data show that TOP2A in patients with hepatocellular carcinoma (HCC) is the expression of tumor tissue is high expression in normal tissue, the experiment of immunohistochemical results also prove the conclusion, especially in the liver cell cancer III midterm highest expression, show TOP2A in the mid-late hepatocellular carcinoma (HCC) has potential application value in the diagnosis. Our results support the conclusion that TOP2A overexpression by Zhang et al. is not due to the amplification of the TOP2A gene [40], amplification is the most common mutation type of TOP2A gene, but the amplification rate is only 0.39%, which proves that the amplification-based mutation is not related to TOP2A overexpression. Studies by Panvichian et al. also show that the overexpression of TOP2A in HCC tumor tissue is not caused by THE2A gene amplification [41]. These results show that TOP2A is involved in the development of liver cancer. So far, there is no research on TOP2A mechanism. Notably, our study found that TOP2A may play a role in promoting HCC development by participating in progesterone-mediated oocyte maturation pathways, oocyte subtraction division pathways, and so on.

In recent years, a growing number of studies have shown that topoisomerase is closely related to cancer. It has been reported that DNA topological isomers, especially IIA topological isomers, have been shown to be therapeutic targets for anti-cancer and antimicrobial drugs [42]. In addition, Liu et al. reported that MDM4 and TOP2A interact with each other after binding and are mutually up-regulated at the post-translational level, resulting in TOP2A protein stability, p53 pathway inhibition and increased tumor cell proliferation [43]. Zhang et al. found that removing TOP2A from colon cancer cells by transfecting specific small interfering RNA significantly inhibited the proliferation and invasion of cancer cells, indicating that their expression in colon cancer was highly correlated with cancer metastasis [15]. In addition, the high expression of TOP2A can be shown in various relevant research reports to be closely related to poor prognosis of lung cancer, bladder urothelial carcinoma, breast cancer, adrenal cortical carcinoma [25, 44–46] and other cancers. In addition, Cai et al. reported that high expression of TOP2A was associated with poor prognosis of HCC, which is consistent with our findings [47].

Taken together, our study supports TOP2A as a potential new biomarker for the prevention, diagnosis, and treatment of hepatocellular carcinoma. But our research is not enough as an indicator of clinical testing, and more in-depth research and clinical trials are needed. And for HOW TOP2A regulates the process of liver cancer this paper is not clear, the next step we intend to find TOP2A gene regulation of liver cancer control axis, to find out its regulatory mechanism.

## Conclusion

The study in this paper shows that the expression of the TOP2A gene is raised in the human hepatocellular carcinoma family, and its high expression is related to poor prognosis in liver cancer patients. This conclusion was supported by bioinformatics and immunohistochemistry experiments. In addition, further studies are needed to confirm whether TOP2A can be used as a biomarker and potential therapeutic target for HCC.

## Supplementary Information

**Additional file 1:** Associations between TOP2A expression and stage across human cancers in TIMER database.

**Additional file 2:** Expression of TOP2A in most normal tissues in human body in BioGPS database.

## Data Availability

All data generated or analysed during this study are included in this published article [and its Additional files 1 and 2].
