# Supplementary figures and images for: Study on the expression of TOP2A in hepatocellular carcinoma and its relationship with patient prognosis
Source: Cancer Cell Int. 2022 Jan 15;22:29. doi: 10.1186/s12935-021-02439-0 (PMC8761301; doi:10.1186/s12935-021-02439-0)

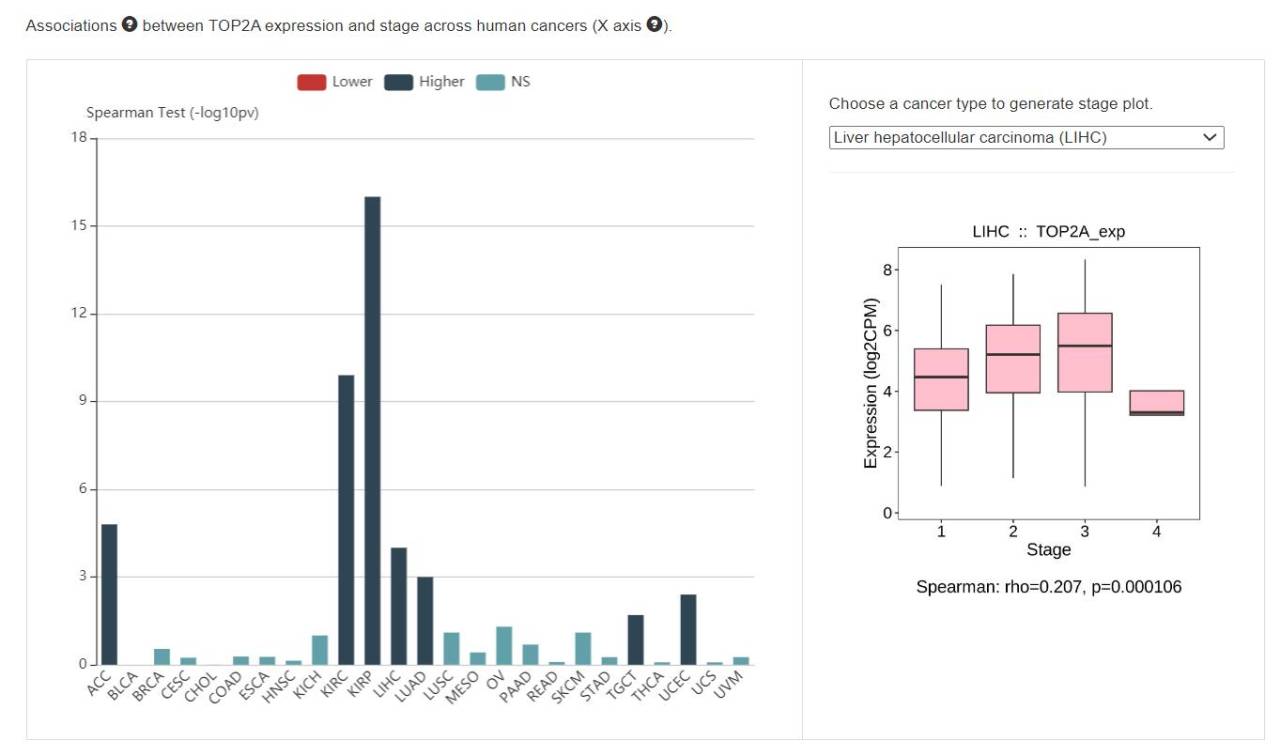

Supplement: Supplementary file 1 — Additional file 1: Associations between TOP2A expression and stage across human cancers in TIMER database. [file 12935_2021_2439_MOESM1_ESM.jpg]

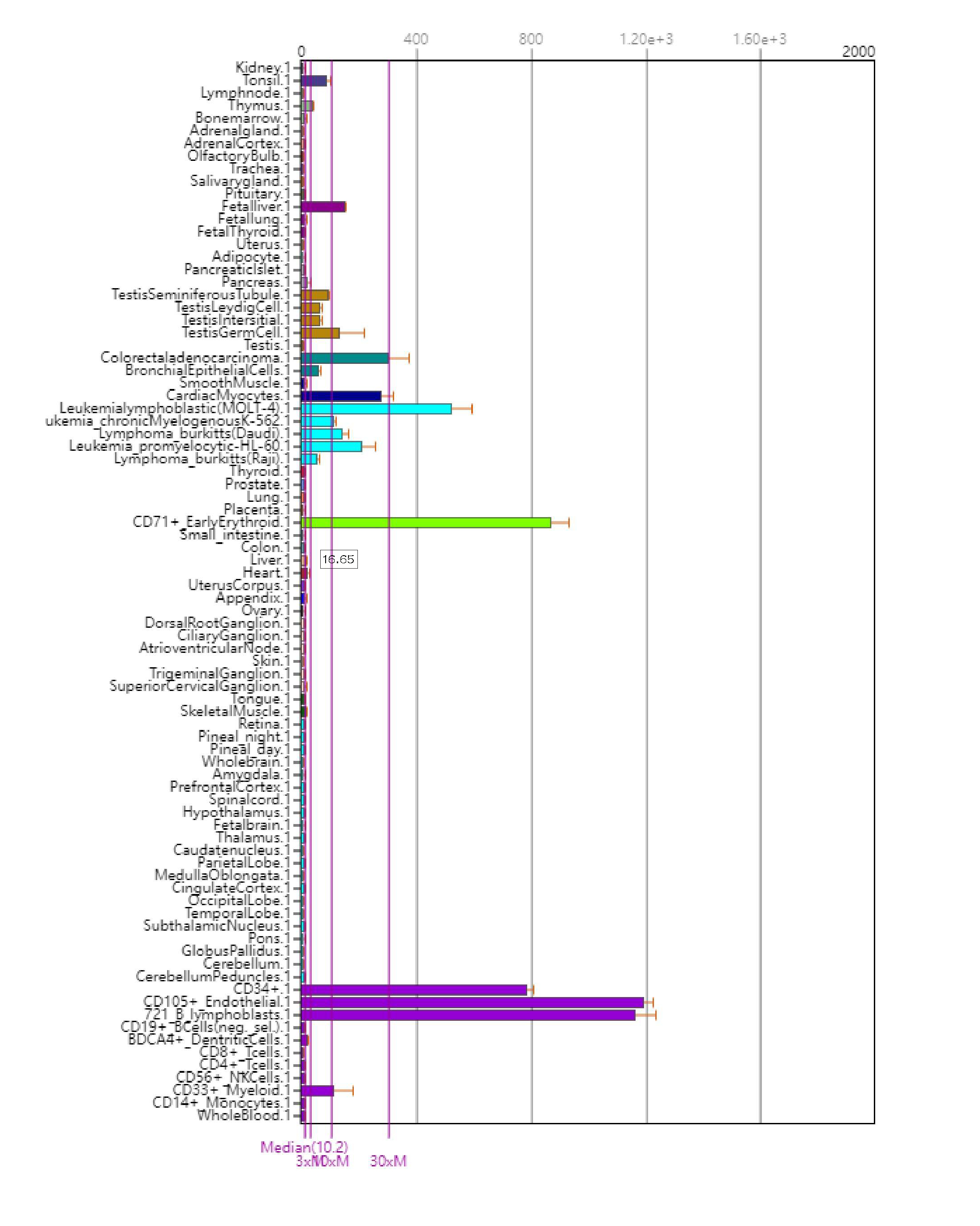

Supplement: Supplementary file 2 — Additional file 2: Expression of TOP2A in most normal tissues in human body in BioGPS database. [file 12935_2021_2439_MOESM2_ESM.jpg]
